# New Appliances and Things Medical

**Published:** 1905-02-11

**Authors:** 


### NEW APPLIANCES AND THINGS MEDICAL.

[We shall be glad to receive at our Office, 28 & 29 Southampton Street, Strand, London, W.C., from the manufacturers, specimens of all new preparations and appliances which may be brought out from time to time.]

### PARKE, DAVIS AND CO.'S NEW PREPARATIONS.

(DETROIT, MICHIGAN, U.S.A.).

WE have received from this firm an adrenalin ointment containing one part of adrenalin chloride in 1,000 parts of ointment base. The preparation is put up in a flexible metal tube. The ointment has a very marked local astringent action and should be useful in all cases where such an action is required. The other two preparations are both solutions for use in this firm's atomisers for oily liquids, the construction of which s familiar to our readers and which produce a very fine spray. The solutions in question are the acetozone inhalant which contains, in addition to acetozone, chloretone (trichlor tertiary butyl alcohol) and liquid paraffin, and may be regarded as antiseptic, anodyne, and emollient in action. The second solution is the adrenalin inhalant containing 1 part of adrenalin chloride in 1,000 parts of neutral oil base, in which is 3 per cent. of chloretone.

### KING'S PREPARED PATENT COOKED OATMEAL.

(ALBION FOOD MILLS, SYCAMORE STREET, LONDON, E.C.).

OATMEAL is distinguished from other cereal meals by containing more proteid, more fat, and more cellulose. From the chemical data alone, it is to be presumed that oatmeal is an extremely nutritious substance. Physiologically, however, the chemical conclusions are not always substantiated, as the excess of cellulose in the case of some individuals leads to imperfect digestion. Several experiments have been made upon digestibility of oatmeal foods. The method being the analysis of the fæces after the ingestion of these foods. From the results of Wolkow, Cholpin, and others, it appears that oatmeal is most digestible when given in the form of well-boiled porridge. Hence the advantage from the standpoint of nutritive value of a pre-cooked and prepared oatmeal. The preparation before us belongs to this class, and it is stated on the label that it only requires a moment's boiling.

### THE TROMMER COMPANY, LIMITED, DIASTASIC EXTRACT OF MALT COMBINED WITH COD-LIVER OIL.

(THE TROMMER COMPANY, LIMITED, 27 CHARTERHOUSE SQUARE, E.C.).

THIS substance consists of a simple mixture of extract of malt and cod-liver oil, containing 60 per cent. of the former and 40 per cent. of the latter. The malt extract contains some active diastase and can therefore be used as an aid to amylaceous digestion as well as a food preparation. In a recent notice on cod-liver oil, and in one on malt extracts we discussed the properties of these substances. The admixture of the two has proved itself to be a therapeutic success, and the preparation before us is as good of its kind as any on the market.

